# Association of single-nucleotide polymorphisms in *RHOB *and *TXNDC3 *with knee osteoarthritis susceptibility: two case-control studies in East Asian populations and a meta-analysis

**DOI:** 10.1186/ar2423

**Published:** 2008-05-10

**Authors:** Dongquan Shi, Takahiro Nakamura, Masahiro Nakajima, Jin Dai, Jianghui Qin, Haijian Ni, Yong Xu, Chen Yao, Jia Wei, Baorui Liu, Shiro Ikegawa, Qing Jiang

**Affiliations:** 1 The Center of Diagnosis and Treatment for Joint Disease, Drum Tower Hospital Affiliated to Medical School of Nanjing University, 321 Zhongshan Road, Nanjing 210008, Jiangsu, China; 2 Laboratory for Bone and Joint Diseases, Model Animal Research Center, Nanjing University, Zhongshan Road 321, Nanjing 210061, Jiangsu, China; 3 Laboratory for Mathematics, Premedical Course, National Defense Medical College, 3-2 Namiki, Tokorozawa, Saitama, 359-8513 Japan; 4 Laboratory for Bone and Joint Diseases, Center for Genomic Medicine, RIKEN, 4-6-1 Shirokanedai, Minato-ku, Tokyo 108-8639, Japan; 5 Department of Oncology, Drum Tower Hospital Affiliated to Medical School of Nanjing University, Zhongshan Road 321, Nanjing 210008, Jiangsu, China

## Abstract

**Introduction:**

Conflicting findings on the association of single nucleotide polymorphisms (SNPs) in *RHOB *and *TXNDC3 *with susceptibility to knee osteoarthritis (OA) have been reported in European Caucasians. To examine the associations of these SNPs with OA in East Asian populations and to evaluate their global significance, we conducted two case-control studies in 955 Chinese and 750 Japanese patients.

**Methods:**

We genotyped the previously implicated SNPs rs585017 (in *RHOB*) and rs4720262 (in *TXNDC3*) in patients with primary symptomatic knee OA with radiographic confirmation and in matched control individuals, and analyzed their associations. We further conducted a meta-analysis of the study findings together with those of previously reported European studies using the DerSimonian-Laird procedure.

**Results:**

A significant association of *RHOB *with knee OA was observed in male Chinese patients (*P *= 0.02). No significant associations were found for *RHOB *in any other comparisons in the East Asian populations. The association of *TXNDC3 *was replicated in Chinese female (*P *= 0.04) and Japanese (*P *= 0.03) patients, although none of these associations persisted after Bonferroni correction. Significant association (*P *= 0.02 for the allelic frequency) with nonsignificant heterogeneity was found in the East Asian replication study. No significant association was found in any comparison in the meta-analysis for all studies.

**Conclusion:**

Our study replicates the association, previously reported in European Caucasians, of *TXNDC3 *with knee OA susceptibility in an East Asian population.

## Introduction

Osteoarthritis (OA; OMIM [Online Mendelian Inheritance in Man] 165720) is a type of arthritis that is caused by breakdown and eventual loss of the cartilage of synovial joints. OA is the most common type of arthritis, with a high incidence in East Asian populations [1,2]. Epidemiological studies have shown that OA has a strong genetic component, and several susceptibility genes for OA have been identified [3-6].

Marh and coworkers [7] examined regulatory polymorphisms in the 5' regions of the genes that potentially allow for differential expression *in vivo*, and they found positive association for *RHOB *and *TXNDC3 *in European Caucasians living in Germany. *RHOB *belongs to the family of small GTPases, and is constitutively expressed and essential in adult articular chondrocytes, but it is significantly downregulated in OA chondrocytes [7]. RHOB (ras homolog gene family, member B), the protein encoded by *RHOB*, is important in the induction of apoptotic cell death that occurs in response to DNA damage [8], and chondrocytes are known to undergo significant DNA damage in OA [9].*TXNDC3 *encodes a thioredoxin protein, and its transcripts appear to be alternatively spliced in monocytes and chondrocytes, with the chondrocyte-specific transcripts lacking exon 2 [7]. Thus, both are good candidate OA genes. However, a replication study in the UK found no association of *RHOB *and *TXNDC3 *with knee OA, even though the ethnicity and disease ascertainment were basically the same in the two studies [10].

To examine the replication of association between the *RHOB *and *TXNDC3 *SNPs and knee OA susceptibility, we conducted two case-control studies in Han Chinese and Japanese populations and a meta-analysis.

## Materials and methods

A population of 955 Chinese individuals were studied; 469 (323 women and 146 men) were consecutively enrolled patients at the Center of Diagnosis and Treatment for Joint Disease (Drum Tower Hospital, affiliated to Medical School of Nanjing University), and 486 were healthy control individuals (316 women and 170 men), who were enrolled at the Center of Physical Examination. All individuals included in the study were Han Chinese living in and around Nanjing. None dropped out during the study. In addition, a population of 750 Japanese individuals were included; 376 patients (327 women and 49 men) and 374 healthy control individuals (116 women and 258 men) who were living in and around Tokyo were recruited by participating hospitals.

Included were patients with knee OA who not only had definite signs and symptoms of OA but also had radiographic evidence of OA. All patients had pain with rest and/or night pain over 5-month period. Patients with knee diseases such as inflammatory, post-traumatic and post-septic arthritis, and skeletal dysplasia were excluded. Radiographic OA was assessed using the Kellgren-Lawrence grading system [11]. Only patients with Kellgren-Lawrence grades of 2 or higher were included. More than 70% of patients had a Kellgren-Lawrence score of 3 or 4. None of the control individuals had ever exhibited any signs or symptoms of arthritis or joint diseases.

The ages of the patients and the controls (mean ± standard deviation) in the Chinese study were 58 ± 13 (range 32 to 93) years and 57 ± 12 (range 40 to 97) years, respectively. We calculated the body mass index to assess the effect of obesity. The body mass index (mean ± standard deviation) was 25 ± 4 kg/m^2 ^for patients and 24 ± 7 kg/m^2 ^for control individuals. There was no statistically significant difference between the two groups. The ages of the patients and the control individuals (mean ± standard deviation) in the Japanese study were 73 ± 7 years and 63 ± 10 years, respectively.

The study protocol was approved by the ethnical committees of the Medical School of Nanjing University, and single nucleotide polymorphism (SNP) Research Center of RIKEN, and informed consent was obtained from patients and control individuals.

### Genotyping

The *RHOB *SNP rs585017 and *TXNDC3 *SNP rs4720262 [7] were genotyped by the 5-nuclease assay (Taqman) using the Mx3000P Real Time PCR System (Stratagene, La Jolla, CA, USA) or ABI 7700 (Applied Biosystems, Foster City, CA, USA). The primers, probes and reaction conditions are available upon request. Genotyping was conducted by laboratory personnel who were blinded to subject status. A random 5% of the samples were subjected to repeat analysis to validate the genotyping procedures. Two authors independently reviewed the genotyping results, data entry and statistical analyses. The distributions of genotypes in all knee OA and control groups conformed to Hardy-Weinberg equilibrium.

### Statistical analysis

Standard χ^2 ^analysis-of-contingency tables were used to compare genotype and allele distributions of *RHOB *and *TXNDC3 *in the case-control study. Differences in clinical characteristics, age, sex and body mass index between genotypes were tested by Mann-Whitney test, Kruskal-Wallis test, or χ^2 ^test using SPSS 12.0 system software (SPSS Inc., Chicago, IL, USA). We assessed association of rs585017 and rs4720262 with stratifications and Hardy-Weinberg equilibrium using the χ^2 ^test. Software R was used in the meta-analysis. Genotype data from previous reports [7,10] and our own genotype data were analyzed using the DerSimonian-Laird procedure [12], based on the random effects model. Power estimates were calculated using the software R.

## Results

### RHOB

The allelic frequency of the susceptible G allele of rs585017 was similar in Chinese and Japanese individuals, and was low (< 4%), unlike that in European Caucasians (> 20%) [8,10]. No GG genotype was detected in East Asian individuals (Table 1). Positive associations with OA were found in comparisons of genotypes and alleles in Chinese men (both *P *= 0.02; Table 2), but these associations did not persist after Bonferroni correction, and no such association was found in Japanese individuals (Table 2). No significant association was detected in any other comparison in East Asian populations (*P *= 0.28 and *P *= 0.27 in recessive and allele modes, respectively). We had 99% and 63% power to detect odds ratios of 2.1 and 1.5 in our study.

**Table 1:** Genotype and allele frequencies of A/G transition SNP (rs585017) of the *RHOB *gene in Chinese and Japanese populations

Group	Number of individuals	Genotype count (frequency)			Allele count (frequency)	
		
		AA	AG	GG	A	G
Chinese patients with knee OA						
All	469	434 (0.925)	35 (0.075)	0	903(0.963)	35 (0.037)
Female	323	303 (0.938)	20 (0.062)	0	626 (0.969)	20 (0.031)
Male	146	131 (0.897)	15 (0.103)	0	277 (0.949)	15 (0.051)
Chinese control individuals						
All	486	460(0.947)	26 (0.053)	0	946 (0.973)	26 (0.027)
Female	316	296 (0.937)	20 (0.063)	0	612 (0.968)	20 (0.032)
Male	170	164 (0.965)	6 (0.035)	0	334 (0.982)	6 (0.018)
Japanese patients with knee OA						
All	376	350 (0.931)	26 (0.069)	0	726 (0.965)	26 (0.035)
Female	327	303 (0.927)	24 (0.073)	0	630 (0.963)	24 (0.037)
Male	49	47 (0.959)	2 (0.041)	0	96 (0.980)	2 (0.020)
Japanese control individuals						
All	371	343 (0.925)	28 (0.075)	0	714 (0.962)	28 (0.038)
Female	113	103 (0.912)	10 (0.088)	0	216 (0.956)	10 (0.044)
Male	258	240 (0.930)	18 (0.070)	0	498 (0.965)	18 (0.035)

OA, osteoarthritis; SNP, single nucleotide polymorphism.

**Table 2:** Association of the A/G polymorphism of the *RHOB *gene with knee osteoarthritis in Chinese and Japanese populations

Groups compared	AA versus AG			A allele versus G allele		
	
	OR	*P*	95% CI	OR	*P*	95% CI
Chinese study						
All patients (*n *= 469) versus all controls (*n *= 486)	0.70	0.18	0.42–1.18	0.71	0.19	0.42–1.18
Female patients (*n *= 323) versus female controls (*n *= 316)	1.02	0.94	0.54–1.94	1.02	0.94	0.54–1.92
Male patients (*n *= 146) versus male controls (*n *= 170)	0.32	0.02	0.12–0.85	0.32	0.02	0.12–0.85
Japanese study						
All patients (*n *= 376) versus all controls (*n *= 371)	1.10	0.74	0.63–1.91	1.10	0.74	0.64–1.89
Female patients (*n *= 327) versus female controls (*n *= 113)	1.23	0.60	0.57–2.65	1.22	0.61	0.57–2.58
Male patients (*n *= 49) versus male controls (*n *= 258)	1.76	0.45	0.40–7.85	1.67	0.46	0.40–7.85

CI, confidence interval; OR, odds ratio.

### TXNDC3

Frequencies of the susceptible T allele of rs4720262 were different between Chinese (9.7% in control individuals) and Japanese (14.2% in control individuals), which were significantly different from the frequency in the UK control individuals [10] (Table 3). A positive association was found only for Japanese patients versus control individuals in the comparisons of TT versus other genotypes combined (*P *= 0.03) and allelic frequency (*P *= 0.04; Table 4). No significant association was detected after stratification by sex. Because the results were mixed, we performed the meta-analysis for the four studies (first German study [7] and three replication studies) and for replication studies. No significant associations were observed in any comparison. Because marginally significant heterogeneity was detected in these analyses (Table 5), we further examined associations stratified by ethnicity. There was a significant association for *TXNDC3 *in East Asian populations with nonsignificant heterogeneity (*P *= 0.03 for CC versus CT+TT; *P *= 0.02 for C allele versus T allele; Table 5).

**Table 3:** Genotype and allele frequencies of C/T transition SNP (rs4720262) of the *TXNDC3 *gene in Chinese and Japanese populations

Group	Number of subjects	Genotype count (frequency)			Allele count (frequency)	
		
		CC	CT	TT	C	T
Chinese patients with knee OA						
All	469	398 (0.849)	65 (0.139)	6 (0.012)	861 (0.918)	77 (0.082)
Female	323	274 (0.848)	45 (0.139)	4 (0.013)	593 (0.918)	53 (0.082)
Male	146	124 (0.849)	20 (0.137)	2 (0.014)	268 (0.918)	24 (0.082)
Chinese control individuals						
All	486	394 (0.811)	90 (0.185)	2 (0.004)	878 (0.903)	94 (0.097)
Female	316	257 (0.813)	59 (0.187)	0	573 (0.907)	59 (0.093)
Male	170	137 (0.806)	31 (0.182)	2 (0.012)	305 (0.897)	35 (0.103)
Japanese patients with knee OA						
All	376	299 (0.795)	74 (0.197)	3 (0.008)	672 (0.894)	80 (0.106)
Female	327	257 (0.786)	68 (0.208)	2 (0.006)	582 (0.890)	72 (0.110)
Male	49	42 (0.857)	6 (0.122)	1 (0.021)	90 (0.918)	8 (0.082)
Japanese control individuals						
All	374	279 (0.746)	84 (0.225)	11 (0.029)	642 (0.858)	106 (0.142)
Female	116	89 (0.767)	24 (0.207)	3 (0.026)	202 (0.871)	30 (0.129)
Male	258	190 (0.736)	60 (0.233)	8 (0.031)	440 (0.853)	76 (0.147)

OA, osteoarthritis; SNP, single nucleotide polymorphism.

**Table 4:** Association of the C/T polymorphism of the *TXNDC3 *gene with knee osteoarthritis in Chinese and Japanese populations

Groups compared	CC versus others			TT versus others			All genotypes^a^	C allele versus T allele		
	
	OR	*P*	95% CI	OR	*P*	95% CI	*P*	OR	*P*	95% CI
Chinese study										

All patients (*n *= 469) versus all controls (*n *= 486)	1.31	0.12	0.93–1.84	3.13	0.14	0.68–14.40	0.06	1.79	0.26	0.25–1.27
Female patients (*n *= 323) versus female controls (*n *= 316)	1.28	0.24	0.85–1.94	-	-	-	0.04	1.15	0.47	0.78–1.70
Male patients (*n *= 146) versus male controls (*n *= 170)	1.36	0.31	0.75–2.45	1.18	0.88	0.16–8.40	0.55	1.28	0.37	0.74–2.21
Japanese study										
All patients (*n *= 376) versus all controls (*n *= 371)	1.32	0.11	0.94–1.86	0.27	0.03	0.08–0.88	0.05	1.39	0.04	1.02–1.89
Female patients (*n *= 327) versus female controls (*n *= 113)	1.11	0.68	0.67–1.85	0.23	0.08	0.04–1.22	0.22	1.20	0.43	0.76–1.89
Male patients (*n *= 49) versus male controls (*n *= 258)	2.15	0.07	0.93–4.94	0.65	0.69	0.08–5.26	0.13	2.16	0.06	0.96–4.84

^a^CC, CT and TT genotypes were grouped together, and a 2 × 3 contingency table analysis was conducted. CI, confidence interval; OR, odds ratio.

**Table 5:** Meta-analysis: summary of association and heterogeneity of the *TXNDC3 *C/T polymorphism in knee osteoarthritis

Study	CC+CT versus TT				CC versus CT+TT				C allele versus T allele			
	
	Summary			Heterogeneity	Summary			Heterogeneity	Summary			Heterogeneity
	
	OR	95% CI	*P*	*P*	OR	95% CI	*P*	*P*	OR	95% CI	*P*	*P*
All	1	0.47–2.16	0.99	0.09	1.35	0.93–1.96	0.11	0.01	1.21	0.95–1.53	0.12	0.07
Replication	1.13	0.38–3.34	0.82	0.053	1.14	0.89–1.47	0.29	0.14	1.14	0.90–1.43	0.28	0.11
Asian	1.15	0.1–12.92	0.91	0.02	1.32	1.03–1.67	0.03	0.97	1.29	1.03–1.61	0.02	0.51

CI, confidence interval; OR, odds ratio.

## Discussion

In *RHOB*, the minor allele frequency in East Asian individuals was below 0.05 and much lower than that in European Caucasians. We could not detect a definite association with OA in East Asian individuals. A weak association was found in male Chinese when the patients were stratified by sex, but the association did not persist after correction with multiple testing, and the trend of difference was reversed in female patients. Meta-analysis also yielded negative results. More than 80% power at a significance level of 5% is desirable for a 'negative' meta-analysis [13]. Our study had adequate power if we had identified the same odds ratio as the original study [7], but we did not have sufficient power to detect an odds ratio as small as 1.5. This lack of association requires confirmation in further replication studies.

The minor allele frequencies of *TXNDC3 *in East Asian individuals were significantly different from those in UK control individuals. The corresponding German frequency was 13%, which was within the Asian frequency range. It appears that rs4720262 provides a good example for the variability of SNPs between different ethnicities or geographic locations. Weak associations were observed in the Chinese and Japanese populations in some comparisons. These associations did not persist after rigorous correction for multiple testing, and the meta-analysis found no associations in the analyses for all and replication studies; however, the analysis after stratification by ethnicity detected a significant association in the East Asian population. Had the meta-analysis of the East Asian study data found an absence of correlation, then it wold be possible to conclude with confidence that there is no association.

The association of *TXNDC3 *with knee OA was replicated in East Asian individuals, whereas a lack of association for *RHOB *was identified. Ethnic differences have been noted in OA susceptibility genes, especially in minor allele frequency between two genes. The association of *LRCH1 *in European [14] has not been replicated in Asian [15] patients, and the association for *CALM1 *identified in Japanese hip OA [16] has not been detected in UK Caucasian patients [17]. In *ASPN*, frequencies of the susceptible D14 allele differ among European patients and between European and Asian patients [18]. The association of *ASPN *with knee OA is found globally, but the effect of *ASPN *exhibits considerable ethnic differences [19]. There is a clear, global link between *GDF5 *and knee OA, but the effect size differs considerably between Europeans and Asian patients [5,20]. As noted in the *LRCH1 *study [15], the difference in the ascertainment criteria is unlikely to account for the discrepancy. A worldwide association study along with functional studies of the susceptibility SNP should be performed to clarify the significance of *TXNDC3 *as an OA susceptibility gene.

## Conclusion

Our study replicates the association, previously reported in European Caucasians, of *TXNDC3 *with knee OA susceptibility in an East Asian population.

## Abbreviations

OA = osteoarthritis; SNP = single nucleotide polymorphism.

## Competing interests

The authors declare that they have no competing interests.

## Authors' contributions

All authors contributed to the final manuscript. In addition, DQS genotyped the samples and participated in the design and analysis of the study, and MN genotyped the Japanese samples. JD, JQ, NJ, YX, CY and JW evaluated the patients and genotyped Chinese samples, and TN helped with the meta-analysis. BL coordinated the study. QJ and SI supervised the whole study.
